# Birth seasonality and calf mortality in a large population of Asian elephants

**DOI:** 10.1002/ece3.746

**Published:** 2013-09-11

**Authors:** Hannah S Mumby, Alexandre Courtiol, Khyne U Mar, Virpi Lummaa

**Affiliations:** 1 Department of Animal and Plant Sciences, University of SheffieldAlfred Denny Building, Western Bank, Sheffield, S10 2TN, U.K; 2 Department of Evolutionary Genetics, Leibniz Institute for Zoo and Wildlife ResearchAlfred-Kowalke-Strasse 17, 10252, Berlin, Germany

**Keywords:** Birth season, climate, conservation, *Elephas maximus*, offspring mortality, working elephants

## Abstract

In seasonal environments, many species concentrate their reproduction in the time of year most likely to maximize offspring survival. Asian elephants (*Elephas maximus*) inhabit regions with seasonal climate, but females can still experience 16-week reproductive cycles throughout the year. Whether female elephants nevertheless concentrate births on periods with maximum offspring survival prospects remains unknown. We investigated the seasonal timing of births, and effects of birth month on short- and long-term mortality of Asian elephants, using a unique demographic data set of 2350 semicaptive, longitudinally monitored logging elephants from Myanmar experiencing seasonal variation in both workload and environmental conditions. Our results show variation in birth rate across the year, with 41% of births occurring between December and March. This corresponds to the cool, dry period and the beginning of the hot season, and to conceptions occurring during the resting, nonlogging period between February and June. Giving birth during the peak December to March period improves offspring survival, as the odds for survival between age 1 and 5 years are 44% higher for individuals born during the high birth rate period than those conceived during working months. Our results suggest that seasonal conditions, most likely maternal workload and/or climate, limit conception rate and calf survival in this population through effects on maternal stress, estrus cycles, or access to mates. This has implications for improving the birth rate and infant survival in captive populations by limiting workload of females of reproductive age. As working populations are currently unsustainable and supplemented through the capture of wild elephants, it is imperative to the conservation of Asian elephants to understand and alleviate the effects of seasonal conditions on vital rates in the working population in order to reduce the pressure for further capture from the wild.

## Introduction

Seasonal conditions are known to influence postnatal mortality in mammals (Descamps et al. 2008) through direct effects on infant survival (Mumby et al. 2013) or indirectly through its effects on maternal body condition and nutrition quality and availability (Pettorelli et al. 2006). For this reason, in seasonal environments mothers may match their timing of conception and birth to the months or seasons with lower offspring mortality (Stearns 1992). Benefits of seasonal breeding have been clearly shown in many short- and midlived species, which concentrate all their reproductive effort into a short period of time characterized by high resource availability (Ogutu et al. 2010; Schaper et al. 2012). For example, in red deer (*Cervus elaphus*) all females give birth within a 2-month time period from mid-May to mid-July (Clutton-Brock et al. 1987), with temperature variation of just a few degrees during gestation having long-term effects on offspring mortality and reproduction (Albon et al. 1983; Kruuk et al. 1999).

In long-lived species, which produce few offspring with long periods of parental dependence and whose reproductive life spans cover several years or decades, the effects of gestational and early-life environment may be manifested both in the first few months after birth when mortality is highest, and in the long term due to the extended duration of offspring dependency (Caughley 1966). However, few studies have investigated the effects of seasonal variation in early-life environment on either birth timing or offspring mortality in long-lived mammals.

Even long-lived aseasonal breeders such as wild African elephants (*Loxodonta africana*) (Laws et al. 1975; Dublin 1983; Moss 1988), killer whales (*Orcinus orca*) (Robeck et al. 1993), and humans (Moore et al. 1999) can concentrate births into certain periods of the year. However, this pattern is not universal: the chimpanzee (*Pan troglodytes*) (Wallis 2002), for example, breeds throughout the year and exhibit no seasonality of births. Despite the ability of humans to reproduce any time of the year, in the Northern hemisphere more human births occur in the spring months (Lummaa et al. 1998). Season of birth in humans is also linked with both short- and long-term effects on mortality (Moore et al. 1998). However, the seasonal pattern of births in human populations does not always maximize offspring survival, possibly because it is influenced both by biological and cultural factors (Lummaa et al. 1998). These two components could also be relevant in natural animal populations interacting with humans, such as working elephants whose seasonal workloads and rest schedules are set by humans but nonetheless reproduce and feed with little human intervention. Elephants have 16-week reproductive cycles throughout the year, each with 2–3 receptive days (Fowler and Mikota 2006). There is variation in cycle duration (range 12–19 weeks), and more variation between than within individuals (Glaeser et al. 2012).

In elephant species, there is some evidence that wild African elephants from Amboseli (Kenya) experiencing poorer early-life environments showed higher mortality before age 2 years (Moss et al. 2011), and that individuals experiencing two drought years during the first years of life had reduced total life expectancy (Moss et al. 2011). Male mortality was more responsive to poor environmental conditions, due to higher energetic demands of male calves (Lee and Moss 1986). In Kenya, 81% of 1030 recorded births occurred between November and May, indicating clear seasonal variation in births (Moss 2001), but the study did not investigate whether seasonal variation in mortality was associated with seasonal changes in birth rates. Female African elephants that reproduce before the start of the rainy season give birth at the time of maximum resource availability (Wittemyer et al. 2007a,b), but there is conflicting evidence as to whether their offspring are more likely to survive their first year (Dublin 1983). Females reproducing before the rains were also more likely to be dominant, and therefore it is not clear whether differential offspring mortality is attributable to environmental conditions, maternal dominance, or their interaction**.** In summary, previous studies of elephants have not yet fully clarified the relationship between birth timing, offspring mortality and seasonal variation in environmental conditions, and in addition they have primarily focused on wild African elephants. African and Asian elephants are known to differ in reproductive endocrinology with a higher rate of noncycling females (Freeman et al. 2004) and synchronicity of cycling (Weissenboeck et al. 2009) in the former species.

**Figure 1:**
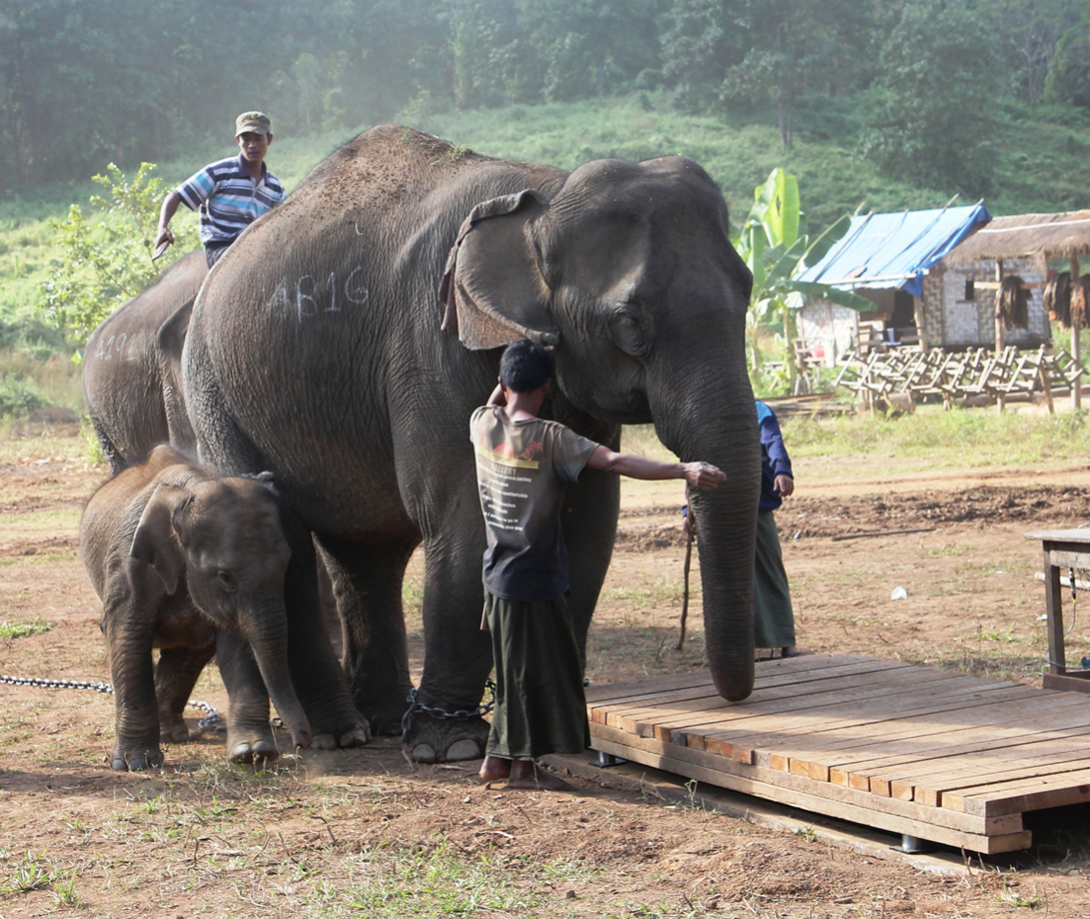
Asian elephant (*Elephas maximus*) female and her calf. Pan Cho Lwin, born on 3 April 1977, is shown with her fourth calf; two older female offspring are still alive, while a male died at age 8 months. Both mother and offspring are being longitudinally weighed and measured as part of the Myanmar Timber Elephant Project. Photograph by Virpi Lummaa.

Asian elephants (*Elephas maximus*; Fig. 1) are characterized by long gestations of around 22 months (Lueders et al. 2012) and an extended infant dependency period of up to 6 years (Fowler and Mikota 2006). They inhabit regions characterized by a seasonal climate, which has been shown to influence mortality rates (Mumby et al. 2013). In addition, this species includes large populations of working elephants that also face the possible effects of seasonal work schedules. They are for this reason a relevant system for studies of effects of seasonal variation on birth timing and short- and long-term mortality of calves, with results that could be applied to management practices. Due to paucity of longitudinal data on Asian elephants, it is not clear whether there is a seasonal pattern of births in wild or captive Asian elephants, or whether females time conceptions or births to the most favorable periods of the year in terms of offspring survival. Two studies of elephants in India found signs of birth seasonality and a peak of births in January: logging elephants in the south (Sukumar et al. 1997), and elephants in a wildlife sanctuary in northwest India (Baskaran et al. 2009). However, the first study miscalculated conception dates (by subtracting 26 instead of 22 months from birth dates), whereas the second includes only 58 individuals. Therefore, further investigation into the links between seasonal conditions, birth seasonality, and calf survival is required.

Here, we investigate seasonal timing of births, stillbirths, and mortality and their relation to birth month and maternal work schedule in a population of semicaptive Asian elephants. We use a unique demographic data set of 2350 working timber elephants from Myanmar born between 1948 and 2000. We investigate (1) the distribution of births through the year, and the effects of climate seasonality (temperature and rainfall) on birth timing. We also analyze (2) the short-term effect of birth month on mortality to age 1 month, during which 10% of calf deaths occur (Mar et al. 2012), (3) the effect of birth month on mortality to age 1 years, a high mortality phase during which calves are completely dependent upon their mothers (Mar et al. 2012), (4) the effect of birth month on mortality to age 5 years, the age at which elephants are weaned and begin the training, and (5) the long-term effect of birth month on mortality to age 17 years, the age at which elephants become full-time working elephants. In all analyses, we test whether effects of birth month on mortality are modulated by sex and birth order. Finally (5) we discuss how such patterns coincide with the known seasonal workload of the mothers.

## Material and Methods

### Study population

Asian elephants are distributed discontinuously across 13 range countries and are classified as endangered on the International Union for Conservation of Nature (IUCN) Red List of threatened species (IUCN 2010). Myanmar maintains the largest population of Asian elephants in the world after India with up to 5000 wild animals, although this figure is debated and could be much lower (Leimgruber et al. 2008), and 5000 semicaptive elephants (Mar et al. 2012). Over half of the semicaptive population is owned by the state through Myanma Timber Enterprise (MTE) and work in the timber industry (Leimgruber et al. 2008). Between-individual variation in workload and rest periods is set by law: all state-owned elephants are subject to the same central government regulations for hours of work per week, working days per year, and tonnage to extract per elephant. For example, in 2010 all mature elephants (17–55 years old) worked 3–5 days a week (depending on weather and forage availability), 5–6 h a day (maximum 8 h) with a break at noon. Based on our study of the logbooks and observation of working elephants, these rules are strictly adhered to.

Throughout the year, elephants are not provisioned and forage unsupervised in the forest at night (Toke Gale 1974). There is no selective breeding and most reproduction takes place at night in the forest where both captive and wild bulls (depending on location) have access to estrus females (Mar 2007). For this reason timber elephants are characterized as semicaptive. Pregnant females are given a rest period from midpregnancy (around 11 months into gestation) until the calf is 1 year old (Toke Gale 1974), although they are tracked by their mahouts (individual caretakers and riders) throughout this period and the calf is monitored. Following this break, mothers are used for light work but are kept with calves at heel and able to suckle on demand. Weaning age is around 4 years, and at around 5 years calves are separated from the maternal herd, tamed and given a name, ID number, and a mahout. Calves are then trained and used for light work duties, until they are put in the workforce at the age of 17 years as logging elephants, continuing to work until retirement at the age of 55 years (Mar 2007).

The Extraction Department of MTE collects and maintains records of all individual elephants. This comprehensive countrywide system is unique to Myanmar and is the equivalent to studbooks kept by Western zoos. Data for each individual include registration number, sex, maternal identity, birth and death dates, location of birth, origin (wild caught or captive born), capture method (if applicable), year of capture and birth dates, identities, and cause of death (if applicable) of offspring. Births of captive-born elephants are recorded precisely. In order to check the health, condition, and working ability of each elephant, individual logbooks are updated by local veterinarians and regional extraction managers at least bimonthly. The multiple sources of data recorded by the MTE (individual elephant logbooks, annual extraction reports, and end-of-the-year reports from each region) allow effective cross-checking of any apparent errors. Our complete data set collected from the records of the MTE contains details of over 8000 elephants from 11 regions (Clubb et al. 2008; Mar et al. 2012; Robinson et al. 2012; Mumby et al. 2013). The sample in this study is a subset of 2350 captive-born elephants (1164 females, 1186 males) with known birth dates, maternal identity, and mortality status (dead or alive by age); 236 of which died by age 1 month, 115 between the ages of 1 month and 1 year, 384 between ages 1 and 5 years, and 239 between the ages of 5 and 17 years. For a further subset of 829 elephants born between 1965 and 2000 in the regions of Gangaw, Katha, Mawlaik, and Shwebo, climatic conditions in the month of birth were available from the Department of Meteorology and Hydrology of Myanmar (for further details of this subset, see Mumby et al. 2013). These data highlight the three clear seasons; from March to June the hot season is characterized by high temperatures (with the highest recorded at 34.4°C in May 1979) and no rainfall (0 mm per month), the monsoon season from July to October has very high rainfall levels (up to 906 mm in September 1997) and high temperatures, finally the cool season from November to February has average monthly temperatures as low as 14.2°C in January 1969 and low rainfall. Individuals that were alive at the end of the study (*N* = 1553) or whose death could not be confirmed (*N* = 3) were censored, that is, that they were considered in our analyses until the age for which their survival status is unknown.

### Statistical analyses

#### Birth seasonality

To test for an effect of month on probability of birth, we implemented a logistic regression model (*N* = 2350 elephants). Each row in the data set was replicated 12 times (one for each possible month) and for each elephant the response variable was “1” for the birth month and “0” for the other 11 months. As such, birth seasonality corresponds to a discrete time event analysis in which row replicates do not introduce pseudoreplication issues (Allison 1982). The following fixed effects were included in the model: month (factor with 12 levels), sex (binary; previous studies show that males may be more sensitive to factors affecting mortality), and birth order (binary coding of firstborn “1” and later born “0”; used to account for differences in birth order and in maternal dominance, as parous females are more likely to be dominant and timing of birth may be associated with maternal dominance; see Moss et al. 2011). For each elephant the cumulative probability of being born in one of the 12 months is 1. Therefore, in our model the intercept is only influenced by the factor month and by effects interacting with it; no other effect modifying the intercept is considered. Sex and birth order were hence not modeled as first-order terms, but only through their interactions with month. As month is a categorical variable, introducing random terms is not justified either, and therefore only fixed effects were considered. In this model it is not possible to control for cohort, location, or kinship effects as interactions of these categorical variables with the covariate month would imply the estimation of different coefficients for each year–month, location–month, or family–month combination, which would produce a largely overparameterized model. We fitted interactions between month and sex, month and birth order, and three-way interaction between month, sex, and birth order. We also repeated the analyses for a subset of 829 elephants for which data on climate in the year of birth were available, and instead of month, probability of birth was then predicted by total monthly rainfall (linear and quadratic terms) and average monthly temperature (linear and quadratic terms). The mean average monthly temperature was 24.9°C (range 16–34°C) and the mean total rainfall was 102 mm (range 0–732 mm).

#### Effect of birth month on mortality

We examined mortality in the first month of life (*N* = 2350, including 101 stillborn), between 1 month and 1 year of age (*N* = 2114), between age 1 and age 5 years (*N* = 1887), and between age 5 and age 17 years (*N* = 866) by birth month using a logistic regression framework. In contrast to the birth seasonality model above, each row of the data set corresponds to a different elephant because only two possible outcomes characterize each elephant (dead or alive). Logistic regression models for each of the four age intervals were implemented as a generalized linear mixed-effect model to predict the probability of death (binary) as a function of three fixed effects: birth month (12 levels), sex (two levels), and birth order (firstborn or later born). We included all two-way interactions between birth month, sex and birth order, and the three-way interaction. We also included three additional random terms to take into account sibship effects (maternal ID, a factor with 1103 levels), location (11 levels), and cohort effects (year of birth, 43 levels).

All analyses were conducted using *R* 2.15.0 (R Development Core Team 2011). Birth models were fitted using the function *glm*, and mortality models were fitted using *glmer* from the package *lme4* version 0.999375-42 (Bates et al. 2011). Test of seasonality effects, using the function *anova*, were performed as a comparison between the deviance of models described above and the same models fitted without the variable month.

## Results

### Birth seasonality

**Figure 2:**
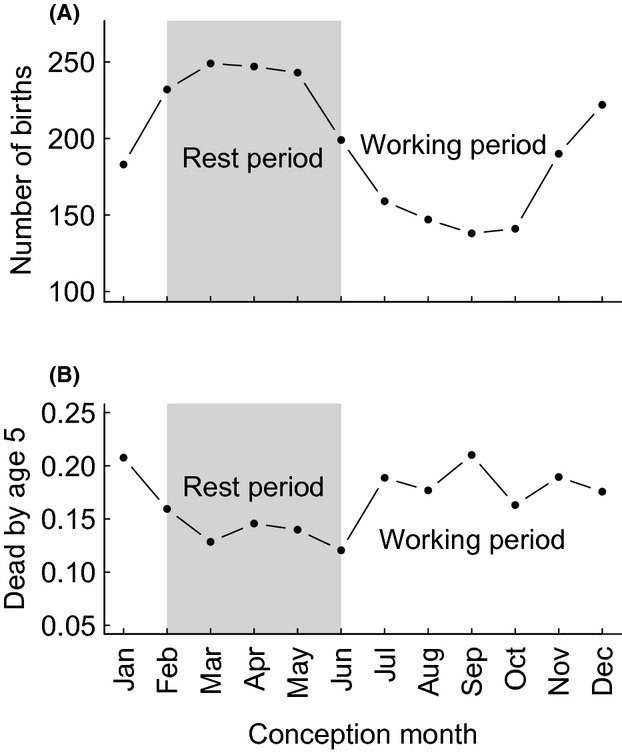
Total number of births (A) and probability of death by age 5 years (B) by birth and conception month 1940–2000 in Asian elephants from Myanmar. Both month of birth and estimated month of conception are displayed. Month of conception is estimated by subtracting 22 months (the average pregnancy duration in Asian elephants) from the date of birth recorded in logbooks. Death by age 5 years is the number of deaths occurring between ages 0 and 5 years divided by the total number of births for each month. The shaded gray region indicates the rest period which all elephants are mandated to take from February to June each year. In total, 2350 births and 735 deaths were recorded.

**Figure 3:**
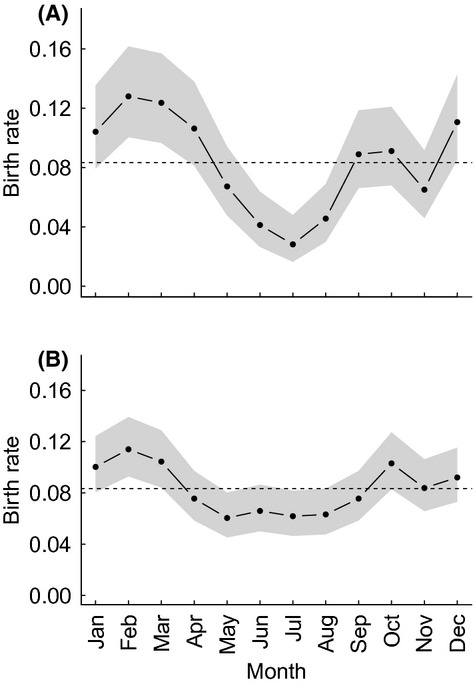
Predictions of monthly probability of birth-by-birth order in Asian elephants from Myanmar. (A) Firstborn calves (*n* = 893) and (B) later-born calves (*n* = 1457). Points indicate the predicted probability from the model and shaded area represents the 95% confidence interval on parameter estimates. The dashed line indicates the predicted birth rate if births were distributed evenly across months. Predictions are for males (similar pattern is observed in females).

We found clear evidence that although female Asian elephants can cycle throughout the year, their reproductive rate fluctuates seasonally. Number of births for all timber sites combined varied between months (*N* = 2350, χ^2^_11_ = 114.15, *P* < 0.0001, Fig. 2A). The months in which more births occurred than would be expected in the absence of seasonality were December, January, February, and March (for each of these 4 months: unilateral Fisher exact tests: all *P* < 0.04; the 4 months pooled together: Fisher exact test: *N*_observed_ = 971, *N*_expected_ = 783, *P* < 0.0001). The months in which fewer births occurred than expected were May, June, July, and August (for each of these 4 months: unilateral Fisher exact tests: all *P* < 0.024; the 4 months pooled together: Fisher exact test: *N*_observed_ = 585, *N*_expected_ = 783, *P* < 0.0001). Accordingly, birth rate was significantly associated with the factor month in our time event analysis (likelihood ratio test or LRT, χ^2^_47_ = 170, *P* < 0.0001). We predicted birth rates controlling for sex and birth order, and identified that, similar to raw data, the predicted birth rate was the highest between December and March. Additionally, there was also a significant interaction between month and birth order (LRT, χ^2^_12_ = 29.4, *P* = 0.0035; Table S1, Fig. 3), indicating that generally, the seasonality of births was most pronounced for firstborn calves. Firstborn individuals represent 38.0% of the population, and 40.9% of firstborns were the sole offspring a mother contributed to this data set. For firstborns, the odds of being born between December and March were 3.17 higher than the odds of being born between May and August, although both periods cover 4 months and the equivalent figure for later-born individuals was 1.69. In contrast, there was no significant interaction between month and sex, indicating that overall the seasonality of births was similar for both male and female calves (LRT, χ^2^_12_ = 15.2, *P* = 0.23). The interaction between month and birth order did not differ between sexes as demonstrated by a nonsignificant triple interaction (LRT, χ^2^_12_ = 11.4, *P* = 0.49).

We also investigated the direct effects of temperature and rainfall on birth rate, which are highly seasonal in Myanmar with three distinct seasons (see Material and Methods). Both temperature and rainfall significantly predicted the probability of births (LRT, χ^2^_47_ = 170, *P* = 0.0006, Table S2). This model also predicted the highest birth rates between December and March corresponding to the cool, dry season and the beginning of the hot season, although birth months were only considered indirectly in the model through associated climatic conditions (see Fig. S1 for details).

### Effect of birth month on mortality

We then investigated whether the observed seasonal timing of births maximizes immediate calf survival prospects and if mothers are timing their births to the most favorable periods. Overall, 10.0% of calves died during the first month, but neither birth month nor the interaction between birth month and birth order or sex modified this risk significantly (LRT, χ^2^_44_ = 45.8, *P* = 0.40). A further 5.4% of calves died between ages of 1 month and 1 year, but again there was neither direct association between mortality and birth month, nor significant interactions between birth month and birth order or sex (LRT, χ^2^_44_ = 36.0, *P* = 0.80).

Finally, we investigated the long-term consequences of the seasonal timing of birth on mortality risk. A total of 18.1% of calves surviving to age 1 year died before reaching independence at age 5 years. This mortality rate was marginally associated with birth month (LRT, χ^2^_44_ = 58.9, *P* = 0.066, Table S3) independently of sex (LRT of the interaction sex:month, χ^2^_23_ = 25.6, *P* = 0.32), but depending on birth order (LRT of the interaction birth order:month, χ^2^_23_ = 35.6, *P* = 0.045) with the mortality of firstborn calves being more influenced by seasonality that later borns. Controlling for birth order and other covariates (considered as fixed or random effects), the lowest calf mortality rates predicted from this model corresponded to being born in the months December–April (Fig. S2) and the same result can directly be observed in raw data (Fig. 2B). The relative similarity with the results from the models of birth seasonality justifies a post hoc analysis where the same model is run with a binary variable indicating whether individuals are born during the 4-month period of high birth rates (*N* = 1173) or not (*N* = 1177), instead of using the variable birth month with 12 different levels. As such, the overall effect of birth season on probability of death became much clearer (LRT, χ^2^_4_ = 16.2, *P* = 0.0028). The odds of survival were 1.44 times higher for calves born during the high birth rate period compared to those born during the low birth rate period. A total of 16.9% of those born in the high birth rate period died between the ages of 1–5 compared to 22.7% of those born in the low birth rate period. For the 27.6% of juveniles which died between 5 and 17 years of age, mortality was no longer associated with birth month (LRT, χ^2^_44_ = 46.3, *P* = 0.38).

## Discussion

Both the presence and the pattern of birth seasonality vary widely across long-lived species. We investigated whether Asian elephants exhibit seasonal variation in births and whether birth seasonality is associated with variation in infant and subadult mortality rates in a population of working elephants. We found strong evidence for seasonal variation in births, with birth number peaking from December to March and showing their lowest levels from May to August. These 4-month periods, respectively, included 41% and 24.8% of the 2350 birth events recorded. Reproducing during this peak birth period led to better calf survival between ages 1 and 5 years. These findings help to design improved management strategies of the working elephant population, and have general relevance for conservation of Asian elephants given that because of its low birth rate and high calf mortality risk, the semicaptive working population is supplemented by capturing endangered wild elephants.

We observed a higher birth seasonality among firstborn individuals. We included birth order in the analyses because mortality of firstborn calves was shown to be higher than of later-born counterparts in Myanmar elephants (Mar et al. 2012) as well as other species such as mountain lions (*Puma concolor*) (Jansen and Jenks 2012), and also because it is a proxy for maternal dominance: later offspring are born to older mothers, and both age and parity are associated with female dominance. Seasonal factors affecting conceptions seem to have a more pronounced effect in nulliparous females, or alternatively, firstborn individuals may be more vulnerable to seasonal factors. It is also possible that those mothers with only a single offspring are of lower “quality” in general, and thus there may be more such mothers among the primiparae; in this data set, 365 females of a total of 1103 included mothers contributed only a single firstborn offspring into the data set. In contrast, sex has no effect on the association between month and probability of birth. This differs from previous reports showing that African elephant males show higher infant mortality under extreme climatic conditions (Moss et al. 2011); however, the lack of sex effect is consistent with our own studies on survival by climatic conditions across all ages in Asian elephants (Mumby et al. 2013).

Given such clear seasonal variation in birth rate, we investigated whether the seasonal timing of birth could be maximizing calf survival prospects in the short or long term, and if mothers are timing their births to the most favorable periods for calf mortality risk. Our results showed that birth month is associated with seasonal variation in mortality of calves aged between 1 and 5 years, with odds ratios for survival being 44% higher for calves born between December and March compared to those born between May and August. The lack of association between birth month and mortality in individuals in other age categories could mean that mortality at those ages is determined by other factors including developmental factors for younger calves (Levitis 2011) or current seasonal conditions for postindependence juveniles (Mumby et al. 2013). Also, before the period between 1 and 5 years, conditions faced by calves may be buffered by maternal care, whereas between the ages of 1 and 5 years the occurrence of weaning exposes offspring to the effects of unfavorable conditions. At the age of 5 years, an increase in mortality was observed in this population (Mar et al. 2012) most likely due to taming. The lack of association in the age 5- to 17-year category could represent selective disappearance of weaker individuals before the age of 5 years (Nussey et al. 2011). Our analyses do not preclude delayed fitness effects of birth seasonality manifested through survival and/or reproduction beyond the age of 17 years (Lee et al. 2013).

We suggest that the factors determining the seasonal pattern of conceptions and number of pregnancies carried to term are either the schedules of work and rest periods determined by humans or seasonal climatic variation, through their effects on stress levels, maternal condition, reproductive cycling, access to mates, adaptation to maximize offspring survival, or a combination of those processes. It is difficult to tease apart these effects in the population as all elephants have the same working pattern and experience very similar climatic variation; in fact, the work undertaken by elephants is determined by the specific climatic conditions at that time of year (Zaw 1997). Asian elephants are the only endangered mammal used as a draught animal. At the beginning of the rainy season (June–August), elephants are primarily used for “aunging” (log pushing). In the late part of the rainy season (September–October), logs often form jams in rivers, these are cleared by elephants through “yelaiking,” an operation that may cause injuries including leg fractures and joint dislocation and is arguably the most stressful work-related activity for timber elephants (Mar 2007). From November to January, elephants drag logs left behind along the river banks. All elephants finish their work season by mid-February, and are moved to rest camps for the hottest and driest part of the year until work resumes around mid-June. Taking a pregnancy duration of 22 months, the resting period corresponds exactly to the 4 months with the highest number of conceptions we identified (December–March), whereas the lowest number of conceptions (May–August) correspond to the period of heavy workload. Elephants conceived in the rest season also experience higher survival, potentially reflecting that the elephants have benefited from the resting period, or that the rest period defined by humans and based on the environment matches the optimum conception or delivery time for elephants. The former explanation appears to be more likely based on our finding that early mortality is not seasonal and previous results indicating that the hot, dry months that characterize the rest period are not ideal conditions for elephants; highest elephant survival is found instead at intermediate temperatures of around 26°C and high rainfall (Mumby et al. 2013).

There are several possible reasons behind higher conception rates during the rest period. In principle, seasonal variation in conception rate can be passive (determined by variable access to mates) or active (with changes in likelihood of females coming into estrus). On one hand, females may have more opportunities to interact with potential reproductive mates (which in the case of Myanmar elephants include both wild and captive males) due to increased time spent in the forest. On the other hand, workload may actively affect reproductive cycling of females, which was shown to be disrupted under poor or stressful conditions (Schneider 2004). Studies of the reproductive cycle of African (Wittemyer et al. 2007a,b) and Asian elephants (Thitaram et al. 2008) indicate that part of the typical 16-week duration of the estrus cycle is extended in poor climatic conditions. In Asian elephants living in a comparable climate, such extension is primarily driven by a prolonging of the luteinizing stage of the estrus cycle, leading to delay of ovulation, by around 10 days on average in the wet season (Thitaram et al. 2008). Postponing conception may avoid the high energetic cost of reproduction until conditions improve (Canale et al. 2012). In this population of timber elephants, we propose that this could imply a delay of conceptions from work season into the rest season, although a 10-day delay of ovulation alone is not sufficient to do so.

Another explanation of the observed birth pattern is that it reflects seasonal variation in the number or proportion of pregnancies carried to full term. Even if conception rate is constant through the year, the same maternal and environmental factors described above may be causing an increase in rates of abortion during the work season. However, early-term abortions (known to frequently occur in Asian elephants in zoos; Lueders et al. 2010) are not recorded in the logbooks and therefore this possibility could not be directly tested. An indirect test for differences in the probability of pregnancies being carried to full term would involve an analysis of seasonal variation in number of stillbirths. A total of 101 stillbirths were included in this analysis and appear to show some variation across months (Table S4), but unfortunately the sample is too small for robust investigation.

Our findings have relevance for conservation of Asian elephants because the working population of elephants in Myanmar represents a large population of an endangered large mammal that, despite having considerably better survival rates than Asian elephants in Western zoos (Clubb et al. 2008), is currently not viable because of consistently too low birth rates and high calf mortality rates. On a wider scale, captive and wild populations of elephants are declining worldwide (Sukumar 2006) and zoo populations may not be sustainable (Faust et al. 2006; Clubb et al. 2008). The semicaptive working population is boosted by capturing wild elephants, a strategy that is costly to the timber industry because they are more difficult to train (Lair 1997) and contributes to the decline in the wild elephant population. Supplementing captive populations with the capture of wild elephants has been legislated against in many range countries including Myanmar, which limits capture of wild elephants to work in the timber industry to a maximum of 50 per year (Mar 2007). However, elephants involved in human–wildlife conflict can also be taken into captivity and even the capture of elephants at a low level could lead to extinction of the wild population in as little as 100 years (Leimgruber et al. 2008).

Therefore, breeding of captive elephants has become increasingly important (Kurt et al. 2008), but current programs are challenged by high mortality rates of calves, particularly in Western zoos (Clubb et al. 2008) and by low birth rates among the logging elephant population (Mar 2007). If the high conception rates reached in the resting season were reached throughout the year, based on birth numbers averages from the 1990s (the most recent decade in the data set), the annual calving rate could rise from 6.9% to 8.8%, meaning increase in the number of calves born from 70 to 88 per year, an increase of 25.7%. The strong implication for management of the semicaptive Asian elephant population is that placing some cycling females on prolonged rest from work may increase fertility rates and bolster the population of Myanmar logging elephants, at the same time protecting the wild population. The cost of having these females on prolonged rest would be countered by the increase in fertility of those females and number of working elephants produced by them.

## Data Accessibility

Data will be archived in Dryad.

## Supporting Information

**Figure S1.** Predictions of monthly probability of birth inferred from climatic data in Asian elephants from Myanmar. Here, in contrast to the model in Figure 3, this model does not explicitly include birth month, but only considered the climatic data at birth (in addition to birth order and sex). The association with birth month was performed a posteriori: For each calf we calculated the predicted probability of birth based on climatic conditions at birth, and then computed the average probability of birth across all calves born in each month. The dashed line indicates the probability of birth if births were distributed equally across months.

**Figure S2.** Predictions of the probability that calves die between 1 and 5 years of age by birth months, in Asian elephants from Myanmar. Predictions were calculated for each calf individually and then averaged across birth months. As such, predictions captured the effect of sex, birth order, relatedness, location, and cohort effects.

**Table S1.** Summary table of model of the effect of month on probability of being born, in Asian elephants from Myanmar. Estimates are expressed on the logit scale. Intercept corresponds to a female later-born elephant born in January. Interaction effects are symbolized with “:.” The model was fitted to observations from 2350 elephants.

**Table S2.** Summary table of model of the effect of climate on probability of being born, in Asian elephants from Myanmar. Estimates are expressed on the logit scale. Intercept corresponds to a female later-born elephant. Interaction effects are symbolized with “:.” The model was fitted to observations from 829 elephants.

**Table S3.** Summary table of model of mortality from age 1 to 5 years in Asian elephants from Myanmar. Estimates are expressed on the logit scale. Intercept corresponds to a female elephant born to a parous mother. Interaction effects are symbolized with “:.” Variance estimates for the random effects were 0.11 for mother identity, 1.52 for region, and 2.45 for the birth year. The model was fitted to observations from 1887 elephants and included a total of 384 deaths.

**Table S4.** Stillbirths by month in Asian elephants from Myanmar. Births with cause of death reported as stillbirth by veterinarians in the necropsy report.

## References

[b1] Albon SD, Guinness FE, Clutton-Brock TH (1983). The influence of climatic variation on the birth weights of Red deer (*Cervus elaphus*. J. Zool.

[b2] Allison PD (1982). Discrete-time methods for the analysis of event histories. Sociol. Methodol.

[b3] Baskaran N, Das S, Sukumar R (2009). Population, reproduction and management of captive Asian elephants (*Elephas maximus*) in Jaldapara wildlife sanctuary, West Bengal, India. Indian For.

[b4] Bates D, Maechler M, Bolker B (2011). http://CRAN.R-project.org/package=lme4.

[b5] Canale CI, Huchard E, Perret M, Henry P-Y (2012). Reproductive resilience to food shortage in a small heterothermic primate. PLoS One.

[b6] Caughley G (1966). Mortality patterns in mammals. Ecology.

[b7] Clubb R, Rowcliffe M, Lee P, Mar KU, Moss C, Mason GJ (2008). Compromised survivorship in zoo elephants. Science.

[b8] Clutton-Brock TH, Major M, Albon SD, Guinness FE (1987). Early development and population dynamics in red deer. I. Density-dependent effects on juvenile survival. J. Anim. Ecol.

[b9] Descamps S, Boutin S, Berteaux D, McAdam AG, Gaillard J-M (2008). Cohort effects in red squirrels: the influence of density, food abundance and temperature on future survival and reproductive success. J. Anim. Ecol.

[b10] Dublin HK, Wasser SK (1983). Cooperation and reproductive competition among female African elephants. Social behavior of female vertebrates.

[b11] Faust LJ, Thompson SD, Earnhardt JM (2006). Is reversing the decline of Asian elephants in North American zoos possible? An individual-based modeling approach. Zool. Biol.

[b12] Fowler ME, Mikota SK (2006). Biology, medicine and surgery of elephants.

[b13] Freeman EW, Weiss E, Brown JL (2004). Examination of the interrelationships of behavior, dominance status, and ovarian activity in captive Asian and African elephants. Zool. Biol.

[b14] Glaeser SS, Hunt KE, Martin MS, Finnegan M, Brown JH (2012). Investigation of individual and group variability in estrous cycle characteristics in female Asian elephants (*Elephas maximus*) at the Oregon Zoo. Theriogenology.

[b15] IUCN (2010). http://www.iucnredlist.org.

[b16] Jansen BD, Jenks JA (2012). Birth timing for mountain lions (*Puma concolor*): testing the prey availability hypothesis. PLoS One.

[b17] Kruuk LEB, Clutton-Brock TH, Rose KE, Guinness FE (1999). Early determinants of lifetime reproductive success differ between the sexes in red deer. Proc. Biol. Sci.

[b18] Kurt F, Mar KU, Garai M, Wemmer C, Christen CA (2008). Giants in chains: history, biology and preservation of Asian elephants in Asia. Elephants and ethics: toward a morality of coexistence.

[b19] Lair R (1997). Gone astray: the care and management of the Asian elephant in domesticity.

[b20] Laws RM, Parker ISC, Johnstone RCB (1975). Elephants and their habitats: the ecology of elephants in Northern Bunyoro, Uganda.

[b21] Lee PC, Moss CJ (1986). Early maternal investment in male and female African elephant calves. Behav. Ecol. Sociobiol.

[b22] Lee PC, Bussière LF, Webber CE, Poole JH, Moss CJ (2013). Enduring consequences of early experiences: 40 year effects on survival and success among African elephants (*Loxodonta africana*. Biol. Lett.

[b23] Leimgruber P, Senior B, Uga M, Aung M, Songer MA, Mueller T (2008). Modeling population viability of captive elephants in Myanmar (Burma): implications for wild populations. Anim. Conserv.

[b24] Levitis DA (2011). Before senescence: the evolutionary demography of ontogenesis. Proc. Biol. Sci.

[b25] Lueders I, Drews B, Niemuller C, Gray C, Rich P, Fickel J (2010). Ultrasonographically documented early pregnancy loss in an Asian elephant (*Elephas maximus*. Reprod. Fertil. Dev.

[b26] Lueders I, Niemuller C, Rich P, Gray C, Hermes R, Goeritz F (2012). Gestating for 22 months: luteal development and pregnancy maintenance in elephants. Proc. Biol. Sci.

[b27] Lummaa V, Lemmetyinen R, Haukioja E, Pikkola M (1998). Seasonality of births in Homo sapiens in pre-industrial Finland: maximisation of offspring survivorship?. J. Evol. Biol.

[b28] Mar KU (2007). The demography and life history strategies of timber elephants in Myanmar.

[b29] Mar KU, Lahdenpera M, Lummaa V (2012). Causes and correlates of calf mortality in captive Asian elephants (*Elephas maximus*. PLoS One.

[b30] Moore SE, Cole TJ, Prentice AM (1998). Hungry season birth predicts excess childhood and adult mortality in rural Gambia. Proc. Nutr. Soc.

[b31] Moore SE, Cole TJ, Collinson AC, Poskitt EM, McGregor IA, Prentice AM (1999). Prenatal or early postnatal events predict infectious deaths in young adulthood in rural Africa. Int. J. Epidemiol.

[b32] Moss C (1988). Elephant memories.

[b33] Moss CJ (2001). The demography of an African elephant (*Loxodonta africana*) population in Amboseli, Kenya. J. Zool.

[b34] Moss CJ, Croze H, Lee PC (2011). The amboseli elephants.

[b35] Mumby HS, Courtiol A, Mar KU, Lummaa V (2013). Climatic variation and age-specific survival in Asian elephants from Myanmar. Ecology.

[b36] Nussey DH, Coulson T, Delorme D, Clutton-Brock TH, Pemberton JM, Festa-Bianchet M (2011). Patterns of body mass senescence and selective disappearance differ among three species of free-living ungulates. Ecology.

[b37] Ogutu J, Piepho H-P, Dublin H, Bhola N, Reid R (2010). Rainfall extremes explain interannual shifts in timing and synchrony of calving in topi and warthog. Popul. Ecol.

[b38] Pettorelli N, Gaillard J-M, Mysterud A, Duncan P, Stenseth NC, Delorme D (2006). Using a proxy of plant productivity (NDVI) to find key periods for animal performance: the case of roe deer. Oikos.

[b39] R Development Core Team (2011). R: a language and environment for statistical computing.

[b40] Robeck TR, Schneyer AL, McBain JF, Dalton LM, Walsh MT, Czekala NM (1993). Analysis of urinary immunoreactive steroid metabolites and gonadotropins for characterization of the estrous cycle, breeding period, and seasonal estrous activity of captive killer whales (*Orcinus orca*. Zool. Biol.

[b41] Robinson MR, Mar KU, Lummaa V (2012). Senescence and age-specific trade-offs between reproduction and survival in female Asian elephants. Ecol. Lett.

[b42] Schaper SV, Dawson A, Sharp PJ, Gienapp P, Caro SP, Visser ME (2012). Increasing temperature, not mean temperature, is a cue for avian timing of reproduction. Am. Nat.

[b43] Schneider JE (2004). Energy balance and reproduction. Physiol. Behav.

[b44] Stearns SC (1992). The evolution of life histories.

[b45] Sukumar R (2006). A brief review of the status, distribution and biology of wild Asian elephants – *Elephas maximus*. Int. Zoo Yearbook.

[b46] Sukumar R, Krishnamurthy V, Wemmer C, Rodden M (1997). Demography of captive Asian elephants (*Elephas maximus*) in Southern India. Zool. Biol.

[b47] Thitaram C, Brown JL, Pongsopawijit P, Chansitthiwet S, Wongkalasin W, Daram P (2008). Seasonal effects on the endocrine pattern of semi-captive female Asian elephants (*Elephas maximus*): timing of the anovulatory luteinizing hormone surge determines the length of the estrous cycle. Theriogenology.

[b48] Toke Gale U (1974). Burmese Timber Elephants.

[b49] Wallis J, Boesch C, Hohmann G, Marchant LF (2002). Seasonal aspects of reproduction and sexual behavior in two chimpanzee populations: a comparison of Gombe (Tanzania) and Budongo (Uganda). Behavioural diversity in chimpanzees and bonobos.

[b50] Weissenboeck NM, Schwammer HM, Ruf T (2009). Estrous synchrony in a group of African elephants (*Loxodonta africana*) under human care. Anim. Reprod. Sci.

[b51] Wittemyer G, Ganswindt A, Hodges K (2007a). The impact of ecological variability on the reproductive endocrinology of wild female African elephants. Horm. Behav.

[b52] Wittemyer G, Rasmussen HB, Douglas-Hamilton I (2007b). Breeding phenology in relation to NDVI variability in free-ranging African elephant. Ecography.

[b53] Zaw UK (1997). Utilization of elephants in timber harvesting in Myanmar. Gajah.

