# Supplementary material for: Birth seasonality and calf mortality in a large population of Asian elephants
Source: Ecol Evol. 2013 Sep 11;3(11):3794–803. doi: 10.1002/ece3.746 (PMC3810875; doi:10.1002/ece3.746)
Supplement: Supplementary file 1 [file ece30003-3794-SD1.docx]

**Table S1 Summary table of model of the effect of month on probability of being born in Asian elephants from Myanmar. Estimates are expressed on the logit scale. Intercept corresponds to a female later-born elephant born in January. The model was fitted to observations from 2350 elephants.** Interaction effects are symbolised with “:”.

|  | **Estimate** | **Std. Error** | **z value** | **Pr(>\|z\|)** | |
| --- | --- | --- | --- | --- | --- |
| (Intercept) | -2.247 | 0.126 | -17.876 | <0.0001 |  |
| February | -0.133 | 0.183 | -0.730 | 0.47 |  |
| March | -0.206 | 0.186 | -1.109 | 0.27 |  |
| April | -0.065 | 0.180 | -0.360 | 0.72 |  |
| May | -0.283 | 0.189 | -1.498 | 0.13 |  |
| June | -0.345 | 0.192 | -1.796 | 0.072 |  |
| July | -0.206 | 0.186 | -1.109 | 0.27 |  |
| August | -0.388 | 0.194 | -1.999 | 0.046 |  |
| September | -0.206 | 0.186 | -1.109 | 0.27 |  |
| October | -0.0483 | 0.180 | -0.269 | 0.79 |  |
| November | -0.0649 | 0.180 | -0.360 | 0.72 |  |
| December | 0.0465 | 0.176 | 0.264 | 0.79 |  |
| January:Male | 0.0526 | 0.176 | 0.299 | 0.77 |  |
| February:Male | 0.330 | 0.177 | 1.866 | 0.062 |  |
| March:Male | 0.303 | 0.183 | 1.660 | 0.097 |  |
| April:Male | -0.193 | 0.191 | -1.012 | 0.31 |  |
| May:Male | -0.214 | 0.210 | -1.016 | 0.31 |  |
| June:Male | -0.0592 | 0.208 | -0.284 | 0.78 |  |
| July:Male | -0.267 | 0.206 | -1.297 | 0.20 |  |
| August:Male | -0.0617 | 0.212 | -0.291 | 0.77 |  |
| September:Male | -0.0517 | 0.196 | -0.263 | 0.79 |  |
| October:Male | 0.131 | 0.177 | 0.741 | 0.46 |  |
| November:Male | -0.0803 | 0.186 | -0.432 | 0.67 |  |
| December:Male | -0.0888 | 0.178 | -0.499 | 0.62 |  |
| January:First born | 0.383 | 0.189 | 2.027 | 0.043 |  |
| February:First born | 0.178 | 0.208 | 0.853 | 0.39 |  |
| March:First born | 0.464 | 0.201 | 2.302 | 0.021 |  |
| April:First born | -0.320 | 0.232 | -1.382 | 0.17 |  |
| May:First born | -0.0651 | 0.236 | -0.276 | 0.78 |  |
| June:First born | -0.0399 | 0.241 | -0.166 | 0.87 |  |
| July:First born | -0.472 | 0.258 | -1.830 | 0.067 |  |
| August:First born | -0.155 | 0.254 | -0.612 | 0.54 |  |
| September:First born | 0.0549 | 0.221 | 0.248 | 0.80 |  |
| October:First born | -0.0437 | 0.213 | -0.205 | 0.84 |  |
| November:First born | -0.437 | 0.240 | -1.820 | 0.069 |  |
| December:First born | 0.0234 | 0.201 | 0.116 | 0.91 |  |
| January:Male:First born | -0.341 | 0.272 | -1.252 | 0.21 |  |
| February:Male:First born | -0.0463 | 0.277 | -0.167 | 0.87 |  |
| March:Male:First born | -0.273 | 0.274 | -0.995 | 0.32 |  |
| April:Male:First born | 0.695 | 0.310 | 2.242 | 0.025 |  |
| May:Male:First born | 0.179 | 0.339 | 0.529 | 0.60 |  |
| June:Male:First born | -0.456 | 0.368 | -1.240 | 0.22 |  |
| July:Male:First born | -0.348 | 0.412 | -0.845 | 0.40 |  |
| August:Male:First born | -0.191 | 0.371 | -0.514 | 0.61 |  |
| September:Male:First born | 0.123 | 0.309 | 0.398 | 0.69 |  |
| October:Male:First born | -0.0924 | 0.294 | -0.315 | 0.75 |  |
| November:Male:First born | 0.164 | 0.333 | 0.491 | 0.62 |  |
| December:Male:First born | 0.181 | 0.281 | 0.645 | 0.52 |  |

**Table S2 Summary table of model of the effect of climate on probability of being born in Asian elephants from Myanmar. Estimates are expressed on the logit scale. Intercept corresponds to a female later-born elephant. The model was fitted to observations from 829 elephants.** Interaction effects are symbolised with “:”.

|  | **Estimate** | **Std. Error** | **z value** | **Pr(>\|z\|)** |
| --- | --- | --- | --- | --- |
| (Intercept) | -1.505 | 1.526 | -0.987 | 0.32 |
| Rain | -0.00202 | 0.000876 | -2.308 | 0.021 |
| Temp | -0.0630 | 0.127 | -0.497 | 0.62 |
| Rain^2^ | 0.00000436 | 0.00000199 | 2.191 | 0.029 |
| Temp^2^ | 0.00121 | 0.00260 | 0.467 | 0.64 |
| First born | 0.866 | 0.369 | 2.350 | 0.019 |
| Male | 0.267 | 0.322 | 0.830 | 0.41 |
| First born:Male | -1.063 | 0.524 | -2.031 | 0.042 |
| Rain^2^:First born | -0.00000083 | 0.00000182 | -0.456 | 0.65 |
| Rain^2^:Male | -0.00000126 | 0.00000174 | -0.726 | 0.47 |
| Temp^2^:First born | -0.00131 | 0.000590 | -2.214 | 0.027 |
| Temp^2^:Male | -0.000357 | 0.000497 | -0.717 | 0.47 |
| Rain^2^:First born:Male | -0.00000167 | 0.00000287 | -0.583 | 0.56 |
| Temp^2^:First born:Male | 0.00172 | 0.000828 | 2.075 | 0.038 |

**Table S3 Summary table of model of mortality from age 1 to 5 years in Asian elephants from Myanmar. Estimates are expressed on the logit scale. Intercept corresponds to a female elephant born to a parous mother. Variance estimates for the random effects were 0.11 for mother identity, 1.52 for region and 2.45 for the birth year. The model was fitted to observations from 1887 elephants and included a total of 384 deaths.** Interaction effects are symbolised with “:”.

|  | Estimate | Std. Error | z value | Pr(>\|z\|) |
| --- | --- | --- | --- | --- |
| (Intercept) | -1.434 | 0.607 | -2.362 | 0.018 |
| February | -0.111 | 0.575 | -0.194 | 0.85 |
| March | -0.399 | 0.611 | -0.653 | 0.51 |
| April | 0.149 | 0.546 | 0.273 | 0.79 |
| May | -0.249 | 0.604 | -0.412 | 0.68 |
| June | 0.0460 | 0.613 | 0.075 | 0.94 |
| July | -0.102 | 0.580 | -0.176 | 0.86 |
| August | 0.0767 | 0.611 | 0.125 | 0.90 |
| September | -0.403 | 0.598 | -0.674 | 0.50 |
| October | -0.116 | 0.577 | -0.201 | 0.84 |
| November | 0.331 | 0.559 | 0.592 | 0.55 |
| December | 0.430 | 0.514 | 0.837 | 0.40 |
| Male sex | -0.222 | 0.558 | -0.397 | 0.69 |
| First born | 0.0546 | 0.571 | 0.096 | 0.92 |
| February:Male | 0.493 | 0.777 | 0.634 | 0.53 |
| March:Male | 0.862 | 0.807 | 1.068 | 0.29 |
| April:Male | 0.521 | 0.794 | 0.657 | 0.51 |
| May:Male | 0.623 | 0.866 | 0.719 | 0.47 |
| June:Male | 0.598 | 0.845 | 0.708 | 0.48 |
| July:Male | 2.014 | 0.807 | 2.495 | 0.013 |
| August:Male | 0.215 | 0.870 | 0.247 | 0.81 |
| September:Male | 1.324 | 0.811 | 1.632 | 0.10 |
| October:Male | 0.558 | 0.793 | 0.704 | 0.48 |
| November:Male | 0.778 | 0.768 | 1.014 | 0.31 |
| December:Male | 0.226 | 0.746 | 0.303 | 0.76 |
| February:First born | 0.0791 | 0.888 | 0.089 | 0.93 |
| March:First born | -0.0542 | 0.911 | -0.060 | 0.95 |
| April:First born | -14.5 | 583.9 | -0.025 | 0.98 |
| May:First born | 0.319 | 0.907 | 0.352 | 0.73 |
| June:First born | 1.007 | 0.902 | 1.116 | 0.26 |
| July:First born | 0.0718 | 0.968 | 0.074 | 0.94 |
| August:First born | -1.061 | 1.125 | -0.943 | 0.35 |
| September:First born | 0.533 | 0.928 | 0.574 | 0.57 |
| October:First born | 0.556 | 0.856 | 0.649 | 0.52 |
| November:First born | 0.677 | 0.879 | 0.770 | 0.44 |
| December:First born | -0.736 | 0.850 | -0.866 | 0.39 |
| Sex:Birth order | 0.0574 | 0.896 | 0.064 | 0.95 |
| February:Male:First born | -0.116 | 1.257 | -0.092 | 0.93 |
| March:Male:First born | -0.617 | 1.290 | -0.478 | 0.63 |
| April:Male:First born | 12.9 | 583.9 | 0.022 | 0.98 |
| May:Male:First born | 0.739 | 1.323 | 0.559 | 0.58 |
| June:Male:First born | -1.962 | 1.464 | -1.341 | 0.18 |
| July:Male:First born | -3.087 | 1.735 | -1.780 | 0.075 |
| August:Male:First born | 1.928 | 1.541 | 1.251 | 0.21 |
| September:Male:First born | -0.134 | 1.290 | -0.104 | 0.92 |
| October:Male:First born | -0.246 | 1.252 | -0.197 | 0.84 |
| November:Male:First born | -1.594 | 1.325 | -1.203 | 0.23 |
| December:Male:First born | -0.206 | 1.270 | -0.162 | 0.87 |

**Table S4 Stillbirths by month in Asian elephants from Myanmar Births with cause of death reported as stillbirth by veterinarians in the necropsy report.**

| **Month** | **Stillbirths** | **Percentage of births stillborn** |
| --- | --- | --- |
| **January** | **9** | **3.6** |
| **February** | **11** | **4.4** |
| **March** | **8** | **3.3** |
| **April** | **10** | **5.0** |
| **May** | **6** | **3.8** |
| **June** | **6** | **4.0** |
| **July** | **7** | **5.1** |
| **August** | **10** | **7.1** |
| **September** | **9** | **4.7** |
| **October** | **6** | **2.7** |
| **November** | **8** | **4.4** |
| **December** | **11** | **4.7** |

**Figure S1. Predictions of monthly probability of birth inferred from climatic data in Asian elephants from Myanmar.** Here, in contrast to the model in Figure 2, this model does not explicitly include birth month, but only considered the climatic data at birth (in addition to birth order and sex). The association with birth month were performed a posteriori: for each calf we calculated the predicted probability of birth based on climatic conditions at birth, and then computed the average probability of birth across all calves born in each month. The dashed line indicates the probability of birth if births were distributed equally across months.

**Figure S2. Predictions of the probability that calves die between 1 and 5 years old by birth months in Asian elephants from Myanmar. Predictions were calculated for each calf individually and then averaged across birth months. As such, predictions captured the effect of sex, birth order, relatedness, location and cohort effects.**
